# The Trump Administration’s ‘earthquake’ is why we need the New Public Health Order

**DOI:** 10.1371/journal.pgph.0005040

**Published:** 2025-09-19

**Authors:** Peg Murray-Evans, Sophie Harman, Maria Birungi Kakinda, Augustina Koduah, Moses Mulumba, Sharifah Sekalala

**Affiliations:** 1 Department of Politics and International Relations, University of York, York, United Kingdom; 2 School of Politics and International Relations, Queen Mary University of London, London, United Kingdom; 3 Afya Na Haki, Nakwero, Uganda; 4 Department of Pharmacy Practice and Clinical Pharmacy, University of Ghana, Legon, Accra, Ghana; 5 School of Law, University of Warwick, Warwick, United Kingdom; PLOS: Public Library of Science, UNITED STATES OF AMERICA

## From earthquake to New Public Health Order

The Trump administration’s ‘earthquake’ [1] on global health cuts across multiple fault-lines: the devastating cuts to aid, curbs on scientific knowledge and expertise, the fracturing of multilateralism, and the ongoing threat of eyewatering tariffs (potentially including trade in pharmaceuticals). These fault-lines are being felt across the world, but most acutely in African countries with aid-dependent health systems. One of the most immediate and damaging effects of US cuts to the HIV response has been widespread stock outs of vital medicines, with the WHO warning that eight countries – six of which are in Africa – could run out of HIV treatments in the coming months [2]. Coming on the back of severe vaccine access constraints during the COVID-19 pandemic, this fresh supply shock compounds the sense that resilient supply chains are a vital component of health equity and security.

**Fig 1:**
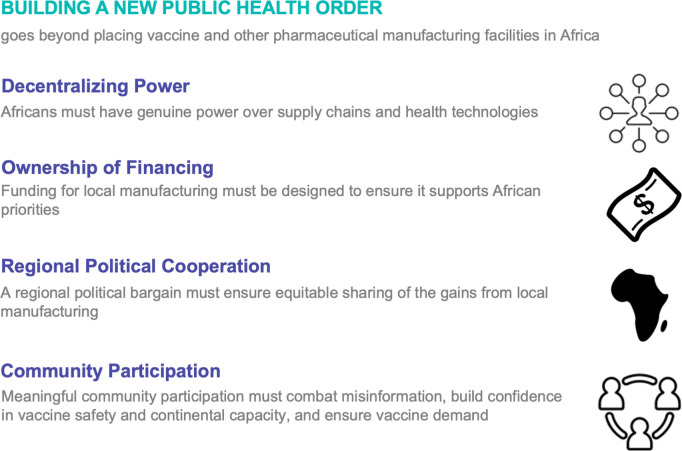
Key requirements to deliver a New Public Health Order.

In this context, Trump’s earthquake is an opportunity to double-down on Africa CDC’s vision for a New Public Health Order, one where Africa is not dependent on foreign aid or the whims of global powers. The main focus of this new order has been rapid investment and interest in the local manufacturing of vaccines and medical counter-measures, to meet Africa CDC’s ambition to make 60% of vaccines for Africa, in Africa [3]. Financing cuts, an emerging trade war, and ensuing supply chain disruption could derail this ambition. To prevent this and really deliver on a New Public Health Order for Africa, we argue that urgent action is required on the decentralisation of power over vaccine and other medical supply chains, financing that supports African health priorities, high-level regional political cooperation, and meaningful community participation (see Fig 1).

## Power over supply chains

Decentralizing power over vaccine technologies is crucial for health security. This was clear to African states during the pandemic, as they called for intellectual property waivers and technology transfers to local manufacturers to challenge the monopoly position of global pharmaceutical firms [4]. This is even more crucial in the face of increasingly inward-looking attitudes to global trade and production in the Global North, not just in the United States but in Europe where EU member states lobbied to keep language on technology transfer in the WHO Pandemic Agreement voluntary [5], and where pharmaceutical companies have called for the strengthening of intellectual property protections in response to Trump’s threatened tariffs [6]. A nascent model has emerged for more equitable sharing of pharmaceutical technologies between small- and medium-sized companies and across national borders in the form of the WHO mRNA Vaccine Technology Transfer Hub based in South Africa. Global pharmaceutical firms have chosen to bypass this initiative, refusing to share technologies and instead proposing their own satellite production facilities in African countries [7]. Yet the opportunity remains for states, Africa CDC, global partners, and local industries to galvanise and incentivise a model of local vaccine production based on genuine transfers of technology and power to Africa.

## Ownership of financing

Africa CDC’s vision for a New Public Health Order emphasises African self-reliance in health financing [8]. African states are taking on some of the financial burden of new investments in vaccine manufacturing capacity by setting up special economic zones and offering tax concessions to pharmaceutical companies. Africa’s intended shift away from donor dependence and development of novel forms of health financing present the opportunity to ensure that funding mechanisms prioritise key societal aims such as health security, as opposed to simply bolstering private profits.

The Pandemic Agreement points the way forward by requiring member countries to develop policies attaching conditions to publicly-funded medical research on issues such as affordable pricing, technology transfer and information sharing [9]. Going beyond this, African states could consider attaching conditions to financing for local vaccine manufacturing initiatives to ensure that they contribute to health equity, sovereignty and self-reliance – in particular by making support conditional on the transfer of technology and knowhow to African manufacturers [10]. The same goes for global partners providing financial support for African vaccine manufacturing, such as through Gavi’s African Vaccine Manufacturing Accelerator and Team Europe’s MAV+ Initiative. To realise the potential of a New Public Health Order, these funding projects must be designed around the priorities identified by African states and regional bodies [11].

## Regional political cooperation

The drive to create a New Public Health Order must increase the impetus for regional health cooperation [12]. Significant strides have been made towards regional pharmaceutical regulatory harmonisation through the creation of the African Medicines Agency and collaboration between the region’s leading national medicines regulatory authorities [13]. The continent is also increasingly galvanizing around the African Continental Free Trade Area (AfCFTA), which offers member countries the chance to develop uniform trade policies under harmonized regulatory frameworks [14]. The AfCFTA aims to eliminate barriers and establish regional vaccine supply chains, supporting Africa’s ambitions for integrated health markets and local manufacturing. On top of this, high level regional political cooperation and solidarity will be essential in realising Africa’s vaccine manufacturing ambitions – even more so in an increasingly hostile geopolitical context. This is vital to ensure that national manufacturing initiatives are coordinated and complementary, and that supply does not outstrip demand [15]. Moreover, since no state can be self-sufficient in vaccine manufacture, strong political partnerships and commitments will be needed to make sure that all countries in the region have a stake in African vaccine manufacturing whether as producers or consumers, both in ordinary times and at times of crisis.

## Community participation for a New Public Health Order

The creation of a New Public Health Order is about much more than simply placing vaccine and other medical manufacturing facilities on the African continent. Africans must have power over supply chains, they must have ownership over financing mechanisms to ensure they support African priorities, and there must be a regional political bargain that ensures the equitable sharing of the gains from local manufacturing. Central to this vision is meaningful community participation, which embodies a whole-of-society approach where communities understand how local manufacturing advances health outcomes. This engagement is vital to combat misinformation, and build confidence in vaccine safety and continental capacity, as well as ensuring vaccine demand. Public trust can be strengthened through educational campaigns and endorsements by community leaders, skilled healthcare professionals, and trusted voices. These imperatives are more important than ever to hold the continent together in an increasingly hostile geopolitical context.
